# Perinatal mortality in non-western migrants in Norway as compared to their countries of birth and to Norwegian women

**DOI:** 10.1186/1471-2458-13-37

**Published:** 2013-01-15

**Authors:** Zainab Naimy, Jostein Grytten, Lars Monkerud, Anne Eskild

**Affiliations:** 1 Department of Obstetrics and Gynecology, Institute of Clinical Medicine, Akershus University Hospital, Lorenskog, Norway; 2 Department of Community Dentistry, University of Oslo, Oslo, Norway; 3 BI Norwegian Business School, Oslo, Norway; 4 Department of Mental Health, Norwegian Institute of Public Health, Oslo, Norway

**Keywords:** Migrant, Perinatal mortality rate, Perinatal death, Maternal health

## Abstract

**Background:**

A large number of women from countries with a high perinatal mortality rate (PMR) settle in countries with a low PMR. We compared the PMRs for migrants in Norway with the PMRs in their countries of birth. We also assessed the risk of perinatal death in offspring of migrant women as compared to offspring of Norwegian women.

**Methods:**

The Medical Birth Registry of Norway and the Norwegian Central Person Registry provided data on births in Norway during the years 1986 to 2005 among all women born in Norway, Pakistan, Vietnam, Somalia, Sri Lanka, Philippines, Iraq, Thailand and Afghanistan. Information on the PMRs in the countries of birth was obtained from the World Health Organisation (WHO) for the years 1995, 2000 and 2004. Mean PMRs in Norway during 1986–2005 were calculated by mother’s country of birth, and the risks of perinatal death by country of birth were estimated as odds ratios (OR) using Norwegian women as the reference. Adjustments were made for mother’s age, plurality, parity, year of birth and gestational age at birth.

**Results:**

The PMRs for migrants in Norway were lower than in their countries of birth. The largest difference was in Afghan women (97 deaths per 1000 births in Afghanistan versus 24 deaths per 1000 births in Afghan women in Norway), followed by Iraqi and Somali women. As compared with Norwegian women, the adjusted odds ratio (OR) of perinatal death was highest for Afghan (OR 4.01 CI: 2.40 – 6.71), Somali (OR 1.83 CI: 1.44 - 2.34) and Sri Lankan (OR 1.76 CI: 1.36 – 2.27) women.

**Conclusions:**

The lower PMRs for migrants in Norway as compared to the PMRs in their countries of birth may be explained by access to better health care after migration. The increased risk of perinatal death in migrants as compared to Norwegians encourages further research.

## Background

The numbers of international migrants have been increasing over the past decades. With 175 million migrants in 2000, the total number is predicted to rise to 230 million by 2050 [1]. Many migrant women are of childbearing age, and maternal health outcomes in these women have become a key priority for many governments [2]. Reflecting the increased focus on migrant women and their reproductive health, the international research collaboration Reproductive Outcomes and Migration (ROAM) was established in 2004 [3]. The main goal of this collaboration has been to study the relationship between migration and reproductive health. Thus, perinatal health outcomes have been recommended to be reported by country of birth [4].

A recent review of studies on stillbirth and infant death among migrants in industrialized countries found higher mortality in migrants compared to the native population in half of the studies, while in the remaining studies similar or lower mortality was found [5]. In Europe, offspring of non-European migrants were consistently found to have higher perinatal mortality rates (PMRs) than the native population [5]. However, comparison between studies may be difficult as the definition of migrants vary across studies [5,6].

Although the risk of perinatal death is higher in non-Western migrants than in the native population, the risk is likely to be lower than in their countries of birth. The PMRs in migrant women’s countries of birth as compared to the PMRs for offspring of migrant women in their new countries has not, to our knowledge, been systematically reported. Such a difference may be an indirect measure of the effect of the health care services in reducing the PMR.

Norway has one of the lowest PMRs in the world [7] and the public health system provides free access to antenatal and obstetric care to all women. Pakistan, Viet Nam, Somalia, Sri Lanka, Philippines, Iraq, Thailand and Afghanistan have some of the highest PMRs in the world [7]. Women from these countries represent some of the largest non-Western migrant groups in Norway [8], and they give birth to approximately 50% of all infants born to non-Western migrants. We compared the mean PMR for each of these migrant groups in Norway during the years 1986 to 2005 with the PMR in their countries of birth in 1995, 2000 and 2004 [7,9,10]. We also studied the risk of perinatal death in offspring of migrant women as compared to offspring of Norwegian women. Adjustments were made for differences in mother’s age at delivery, plurality, parity, year of birth and gestational age at birth.

## Methods

### Study design

Information on PMRs in Pakistan, Viet Nam, Somalia, Sri Lanka, Iraq, Philippines, Thailand and Afghanistan was obtained from the World Health Organization (WHO) reports on perinatal mortality for the years 1995, 2000, and 2004 [7,9,10]. Mean PMRs in Norway during the years 1986 to 2005 by country of birth were obtained by linkage between the Medical Birth Registry of Norway [11] and the Central Person Registry of Norway [12]. The Medical Birth Registry contains information on all births in Norway, including offspring death before or at birth. This information is compulsorily reported by the health professionals attending the delivery [11]. Information on country of birth and early neonatal death (before seven completed days after birth) was obtained from the Central Person Registry of Norway [12].

### Study population and setting

In order to study risk of perinatal death in offspring of migrant women compared to Norwegian women, we included births after gestational week 22 in Norway during the years 1986 to 2005. If information on gestational age at birth was missing, births of offspring with birth weight above 500 grams were included [13]. A total of 1 108331 births met our inclusion criteria, of which 5364 were excluded because of missing information on vital status in the perinatal period. The excluded births represented less than one percent of the births in each migrant group and in Norwegian women. We included births among non-Western women who were born in Pakistan (11351), Viet Nam (6169), Somalia (5410), Sri Lanka (4933), Philippines (4662), Iraq (3829), Thailand (3204) and Afghanistan (665) and births among Norwegian women (1062744). A woman was defined as Norwegian if she had two parents born in Norway.

### Variables

Perinatal Mortality Rate (PMR) was defined as number of offspring deaths after 22 weeks of gestation and before seven completed days after birth per 1000 births (live and stillborn) [9]. Perinatal mortality comprises both stillbirths and early neonatal deaths; the stillbirth rate is defined as number of fetal deaths after 22 weeks of gestation per 1000 births (live and stillborn), and the early neonatal mortality rate is defined as number of infant deaths within seven days after birth per 1000 births (liveborn) [9]. For births before 1999, gestational age at birth was calculated from the date of last menstrual period, whereas after 1999, gestational age was based on estimation of term date at routine fetal ultrasonographic examination in pregnancy week 17–19 [14].

Mother’s country of birth was coded; Pakistan, Viet Nam, Somalia, Sri Lanka, Philippines, Iraq, Thailand, Afghanistan and Norway. Mother’s age at delivery, plurality, parity, year of birth and gestational age at birth as obtained from the Medical Birth Registry [11], were included as potentially confounding variables in the analyses. Mother’s age at delivery was categorized as < 20, 20–24, 25–29, 30–34 and ≥ 35 years old, plurality was categorized as singleton and multiple births. Parity was categorized as 0, 1, 2 and 3 or more previous deliveries and year of birth as 1986–1990, 1991–1995, 1996–2000 and 2001–2005. Gestational age at birth was categorized as < 37 weeks (preterm) and ≥ 37 weeks of gestation.

### Statistical methods

The mean PMRs for migrants in Norway during the period 1986 to 2005 were calculated and compared with WHO estimates of the PMRs in their countries of birth for the years 1995, 2000 and 2004. The risks of perinatal death in Norway according to mother’s country of birth were estimated as crude and adjusted odds ratios (OR) with 95% confidence intervals (95% CI). Norwegian women were used as the reference. Adjustments were made for mother’s age at delivery, plurality, parity, year of birth and gestational age at birth.

The risks of stillbirth and early neonatal death for offspring of migrant women as compared to offspring of Norwegian women were estimated as crude ORs with 95% CI. The proportions of perinatal deaths that were preterm were calculated in migrant women and in Norwegian women, and differences in proportions were tested by using chi-square test.

## Results

**Figure 1:**
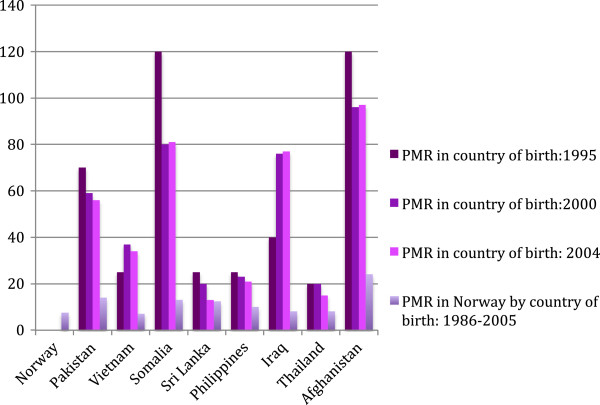
Perinatal mortality rates (PMRs) for migrants in Norway and PMRs in their countries of birth*. *Figures obtained from the World Health Organisation [7,9,10].

The mean PMRs for migrants in Norway during the years 1986 to 2005 were lower than the PMRs in their countries of birth in the years 1995, 2000 and 2004 (Table 1, Figure 1). When using the WHO estimates from 2004, the largest absolute difference in PMR was seen in Afghan women, with 97 deaths per 1000 births in Afghanistan versus 24 deaths per 1000 births in Afghan women in Norway. The second largest difference was for Iraqi women, followed by Somali women. For women from countries with a relative low PMR, such as Sri Lanka, the absolute difference between the PMR in the country of birth in 2004 and the PMR in Norway was small.

**Table 1:** Offspring mortality in migrants in Norway and in their countries of birth*

**Country**	**PMR 1995 in country of birth**	**PMR 2000 in country of birth**	**PMR 2004 in country of birth**	**Mean PMR 1986 – 2005 in Norway**	**Mean stillbirth rate 1986 – 2005 in Norway**	**Mean ENMR 1986 – 2005 in Norway**
**Norway**				7.6	5.0	2.6
**Pakistan**	70	59	56	14.0	9.7	4.3
**Vietnam**	25	37	34	7.1	4.7	2.4
**Somalia**	120	80	81	13.1	8.9	4.3
**Sri Lanka**	25	20	13	12.4	9.1	3.3
**Philippines**	25	23	21	10.1	7.5	2.6
**Iraq**	40	76	77	8.1	4.7	3.4
**Thailand**	20	20	15	8.1	4.7	3.4
**Afghanistan**	120	96	97	24.1	13.5	10.7

Perinatal mortality rates (PMR) in the country of birth of migrant groups living in Norway for the years 1995, 2000 and 2004. Mean PMRs, stillbirth rates and early neonatal mortality rates (ENMR) in Norway by country of birth from 1986 to 2005. * Figures obtained from the World Health Organization [7,9,10].

The PMR for offspring of Norwegian women (7.6 per 1000) was lower than for all migrant groups, except for offspring of Vietnamese women (7.1 per 1000) (Table 1). As compared with Norwegian women, the crude OR for perinatal death was particularly increased in Afghan (OR 3.21 CI: 1.96 – 5.28), Pakistani (OR 1.85 CI: 1.58 – 2.17), Somali (OR 1.73 CI: 1.37 – 2.19) and Sri Lankan (OR 1.63 CI: 1.26 – 2.10) women (Table 2).

**Table 2:** Numbers and odds ratios (ORs) of perinatal death by country of birth, and background factors

**Background factors**	**Perinatal deaths (n) Yes**	**Perinatal deaths (n) No**	**Crude OR (95% CI)**	**Adjusted OR (95% CI)**
**Mother’s country of birth**				
Norway	8097	1054647	Reference	Reference
Pakistan	159	11192	1.85 (1.58 – 2.17)	1.72 (1.46 – 2.02)
Vietnam	44	6125	0.94 (0.69 – 1.26)	0.80 (0.59 – 1.08)
Somalia	71	5339	1.73 (1.37 – 2.19)	1.83 (1.44 – 2.34)
Sri Lanka	61	4872	1.63 (1.26 – 2.10)	1.76 (1.36 – 2.27)
Philippines	47	4615	1.33 (0.99 – 1.77)	1.20 (0.90 – 1.61)
Iraq	31	3798	1.06 (0.75 – 1.51)	1.18 (0.82 – 1.69)
Thailand	26	3178	1.07 (0.72 – 1.57)	1.02 (0.69 – 1.51)
Afghanistan	16	649	3.21 (1.96 – 5.28)	4.01 (2.40 – 6.71)
**Mother’s age at delivery**				
<20	391	37545	1.42 (1.27 – 1.58)	1.14 (1.02 – 1.27)
20-24	1628	221415	Reference	Reference
25-29	2743	396675	0.94 (0.88 – 1.00)	1.07 (1.01 – 1.14)
30-34	2426	308155	1.07 (1.00 – 1.14)	1.25 (1.17 – 1.34)
≥35	1364	130625	1.42 (1.32 – 1.53)	1.53 (1.41 – 1.65)
**Plurality**				
Singleton	7481	1061349	Reference	Reference
Multiple	1071	33066	4.60 (4.31 – 4.90)	1.57 (1.46 – 1.68)
**Parity**				
0	3730	453860	Reference	Reference
1	2597	388417	0.81 (0.77 – 0.86)	0.88 (0.83 – 0.92)
2	1444	182964	0.96 (0.90 – 1.02)	0.95 (0.89 – 1.02)
3+	781	69174	1.37 (1.27 – 1.48)	1.11 (1.01 – 1.21)
**Year of birth**				
1986 – 1990	2545	271793	1.45 (1.37 – 1.55)	1.19 (1.11 – 1.27)
1991-1995	2304	284670	1.26 (1.18 – 1.34)	0.95 (0.89 – 1.01)
1996- 2000	2027	277461	1.13 (1.06 – 1.21)	0.95 (0.89 – 1.02)
2001 – 2005	1676	260491	Reference	Reference
**Gestational age at birth**				
<37 weeks	5671	135321	13.95 (13.33- 14.60)	13.16 (12.56- 13.80)
≥ 37 weeks	2881	959094	Reference	Reference

Numbers and crude and adjusted odds ratios (OR) with 95% confidence intervals (CI) for perinatal death according to country of birth and other study factors in women giving birth in Norway from 1986 to 2005 (n= 1 102967).

Overall migrant women differed from Norwegian women in more often being multiparous, giving birth preterm, being above 35 years of age at delivery, having fewer multiple births and more often giving birth towards the end of the study period (Table 3). After adjustment for potential confounding factors, the OR of offspring death according to migrant groups did not change significantly (Table 2). Also, plurality, parity, mother’s age, year of giving birth and gestational age were factors associated with perinatal death.

**Table 3:** Characteristics of women who gave birth in Norway 1986–2005 by country of birth

	**Pakistan (%)**	**Vietnam (%)**	**Somalia (%)**	**Sri Lanka (%)**	**Philippines (%)**	**Iraq (%)**	**Thailand (%)**	**Afghanistan (%)**	**Norway (%)**
**Number of births**	11351	6169	5410	4933	4662	3829	3204	665	1062744
**Mother’s age at delivery**									
<20	357 (3.1)	158 (2.6)	193 (3.6)	57 (1.2)	61 (1.3)	157 (4.1)	84 (2.6)	22 (3.3)	36847 (3.5)
20-24	3478 (30.6)	1446 (23.4)	1189 (22.0)	844 (17.1)	703 (15.1)	868 (22.7)	634 (19.8)	201 (30.2)	213680 (20.1)
25-29	3979 (35.0)	2201 (35.7)	1836 (33.9)	1835 (37.2)	1498 (32.1)	1336 (34.9)	1059 (33.0)	240 (36.1)	385434 (36.3)
30-34	2353 (20.7)	1560 (25.3)	1438 (26.6)	1542 (31.3)	1483 (31.8)	984 (25.7)	880 (27.5)	135(20.3)	300206 (28.25)
≥35	1184 (10.4)	804 (13.0)	754 (13.9)	655 (13.3)	917 (19.7)	484 (12.6)	547 (17.1)	67 (10.1)	126577 (11.9)
**Plurality**									
Singleton	11087 (97.7)	6082 (98.6)	5276 (97.5)	4821 (97.7)	4579 (98.2)	3713 (97.0)	3123 (97.5)	649 (97.6)	1029500 (96.9)
Multiple	264 (2.3)	87 (1.4)	134 (2.5)	112 (2.3)	83 (1.8)	116 (3.0)	81 (2.5)	16 (2.4)	33244 (3.1)
**Parity**									
0	3372 (29.7)	2334 (37.8)	1332 (24.6)	2147 (43.5)	2153 (46.2)	1371 (35.8)	1283 (40.0)	214 (32.2)	443383 (41.7)
1	2882 (25.4)	1956 (31.7)	1168 (21.6)	1810 (36.7)	1655 (35.5)	1029 (26.9)	1188 (37.1)	160 (24.1)	379166 (35.7)
2	2323 (20.5)	1052 (17.0)	971 (17.9)	779 (15.8)	602 (12.9)	605 (15.8)	518 (16.2)	122 (18.3)	177436 (16.7)
3+	2773 (24.4)	827 (13.4)	1939 (35.8)	197 (3.9)	252 (5.4)	824 (21.5)	215 (6.7)	169 (25.4)	62759 (5.9)
**Year of birth**									
1986-1990	2368 (20.9)	957 (15.5)	135 (2.5)	378 (7.7)	940 (20.2)	50 (1.3)	312 (9.7)	11 (1.6)	269187 (25.3)
1991-1995	2595 (22.8)	1676 (27.2)	762 (14.1)	1114 (22.6)	1010 (21.7)	242 (6.3)	543 (16.9)	76 (11.4)	278956 (26.2)
1996-2000	2959 (26.1)	1717 (27.8)	1569 (29.0)	1771(35.9)	1193 (25.6)	935(24.4)	799 (24.9)	90 (13.5)	268455 (25.3)
2001-2005	3429 (30.2)	1819 (29.5)	2944 (54.4)	1670 (33.8)	1519 (32.6)	2602 (68.0)	1550 (48.4)	488 (73.4)	246146 (23.2)
**Gestational age at birth**									
<37 weeks	1661 (14.6)	1041 (16.9)	642 (11.8)	599 (12.1)	674 (14.5)	406 (10.6)	449 (14.0)	67 (10.1)	135453 (12.7)
≥ 37 weeks	9690 (85.4)	5128 (83.1)	4768 (88.1)	4334 (87.8)	3988 (85.5)	3423 (89.4)	2755 (86.0)	598 (89.9)	927291 (87.3)

There was little difference between migrant and Norwegian women in the prevalence of preterm births; 13.8% (5539/40223) versus 12.7% (135453/1062744), (p= < 0.001, chi square test). In migrants, 65.5% (298/455) of all perinatal deaths occurred in preterm births as compared to 66.4% (5373/8097) in Norwegian women (p= 0.705, chi square test).

The stillbirth rates were higher than the early neonatal mortality rates for offspring of both Norwegian and migrant women (Table 1). In general, offspring of migrants had an increased risk of stillbirth (OR: 1.53 CI: 1.36 – 1.71) and early neonatal death (OR: 1.41 CI: 1.20 – 1.67) as compared to offspring of Norwegian women. The increase in both stillbirth rates and early neonatal mortality rates was true for most of the different migrant groups (Table 1). Differentially increased risk in stillbirth as compared to early neonatal death could not be estimated for any migrant group (data not shown). Thus, for all migrant groups the relative increased risk of stillbirth was similar to that of early neonatal death; however, the numbers in subgroups were small (data not shown).

## Discussion

For non-Western migrants the perinatal mortality rate (PMR) was up to 9 times higher in their country of birth than in Norway (Table 1). The difference in PMR between the country of birth and the host country was largest for offspring of Afghan, Iraqi and Somali women. Nevertheless, the risk of perinatal death was higher in offspring of migrant women than in offspring of Norwegian women. For Afghans the risk of perinatal death was four times higher than in Norwegians.

The first WHO report on perinatal mortality in 1995 initiated improved recording of country specific PMRs [9]. However, the accuracy of reporting and the legal requirements for notification of fetal deaths and live births still vary by country [7,9]. The WHO perinatal mortality estimates cannot be interpreted as precise figures of the PMR in each country. In particular, the PMRs in war affected countries such as Afghanistan and Somalia need to be interpreted with caution. The WHO methodology for estimating PMRs, however, has improved since 1995 [7,9]. Sri Lankan migrants in Norway are mainly Tamils from the conflict affected regions of Sri Lanka [15]. Rates of stillbirths, neonatal and maternal deaths have been reported to be higher in these regions than in other regions of Sri Lanka [16-18]. We used the PMR for Sri Lanka as a whole in our study. The difference in PMR between Sri Lanka and Norway may therefore represent underestimates for the Tamils.

Due to the time consuming process of updating national registries, we lacked complete information on perinatal deaths by country of birth beyond 2005. However, there are no indications that our results should not be valid beyond this time period. To our knowledge, no prior studies have compared PMRs for migrants in their host country with the PMRs in their countries of birth. However, risks of perinatal death in migrants as compared to the native population in the host country have been reported. Refugees and non-European migrants in Europe and also foreign born blacks in the United States have high excess perinatal mortality compared to the native population [5]. Studies from Norway and Sweden report increased risk of perinatal death in offspring of Somali women in particular [5,19]. In the United Kingdom offspring of women born in Pakistan are at high risk [20]. Overall the PMRs in these Western countries, in particular the Scandinavian countries, are among the lowest in the world [7]. We are not aware of prior studies that have reported the PMRs for Sri Lankan, Pilipino, Iraqi, Thai and Afghan migrants from any European country.

Our results suggest that migrants from countries with a high PMR benefit substantially from the health care services in Norway. The difference between the PMR in Norway and country of birth may thus be an indirect measure of the effect of quality health care available to all women in attaining low PMR. However, low PMRs in migrants in Norway may also be explained by other factors that affect women’s health, such as improved housing, sanitation and educational opportunities [21] in Norway.

Despite access to quality health care in Norway, most migrant groups have a higher PMR than Norwegian women. Offspring of migrants also have overall higher stillbirth and early neonatal mortality rates than offspring of Norwegian women. The increased risk of perinatal death in migrants cannot be explained by a disproportional increase in risk of stillbirths or of early neonatal deaths. Furthermore, the small differences between migrant and Norwegian women in the prevalence of preterm births and of perinatal deaths in preterm deliveries do not provide further understanding of the excess perinatal mortality in migrants.

Individual information on migration status, social and behavioral factors that could explain the differences in risk were not available in our data. While diabetes and preeclampsia have been associated with increased risk of perinatal death [19,22], we do not know whether information on maternal diseases in The Medical Birth Registry of Norway is valid for migrant women [22,23]. The reported differences in perinatal mortality between groups in our study should, however, encourage further research on risk factors in these migrant groups.

Some differences between migrants groups that could explain differences in perinatal mortality are discussed below. 

The majority of Afghan, Iraqi, Somali, Vietnamese and Sri Lankan women in Norway are either refugees or have migrated for unification with a family member with refugee status in Norway [24]. Refugees are more likely to have been affected by malnutrition, psychological distress and lack of health care services than people who have been able to plan their migration [25]. In Norway, refugees and their families have substantially worse living conditions than the rest of the population, especially if they are newly settled [26]. As the majority of Afghan, Iraqi and Somali migrants had lived in Norway for less than five years in 2004 [26], we assume that their acculturation process is at a premature stage. Their health seeking behavior and cultural practices concerning pregnancy and childbirth may thus be similar to those in their countries of birth. Cultural practices such as reducing food intake to avoid large sized infants and thereby complicated deliveries, have been reported in Somali women residing in Sweden [27]. The low risk of perinatal death in Iraqi migrants as compared to other migrant groups, may partially be due to a better adaption to the Norwegian health care system, since Iraq had a well-functioning health care system and low PMR in the 1980s [28,29]. Though, after the initiation of the United Nations sanctions against Iraq and the Gulf war in 1990, the PMR increased [29].

Vietnamese women may have fewer barriers in accessing healthcare as the majority have lived in Norway for over 10 years and are well-integrated in the Norwegian society [22]. Also, the low risk of perinatal death in Vietnamese migrants in Norway may be due to their background as political refugees and an advantageous socioeconomic background [30]. Such selection, described as the “healthy migrant effect” may explain the low PMR for some migrant groups in Norway [31].

The majority of Pakistani women migrate to Norway after marrying a man with Pakistani background living in Norway [32]. In Pakistani migrants consanguineous marriages are common and may contribute to 29% of the stillbirths and infant deaths in this group [33]. The lower risk of perinatal death in offspring of Thai and Filipino women compared to other migrant groups may partially be associated with 84–95 % of these women being married to a Norwegian man [32]. This may ease their acculturation process into the Norwegian society, making it easier to pass cultural and communication barriers in accessing health care services.

As perceptions of somatic symptoms may differ by culture [20], there may also be cultural determinants of perinatal care. Non-Western migrants in Norway and in the Netherlands have been found to be less prone to attend the antenatal care program, with fewer numbers of antenatal visits and subsequently poorer detection of complications [34-36]. Furthermore, inadequate communication in perinatal care to non-Western migrants has been reported in Norway, Sweden and the Netherlands [35-38], suggesting that problems in interpretation of clinical symptoms may have been disturbed [38]. Suboptimal factors in perinatal care, such as inadequate medication, insufficient surveillance of intrauterine growth restriction (IUGR) and refusal of Caesarean-sections by mothers has been reported in Somali women in Sweden [37], and may be due to miscommunication. The mechanisms behind the health seeking behavior of migrant women and the cultural framework used by these groups in articulating their symptoms are insufficiently understood.

## Conclusion

The lower perinatal mortality rates (PMRs) for migrants in Norway as compared to their countries of birth, suggest that quality health care is fundamental in perinatal mortality reduction. There is however, an increased risk of perinatal death in many migrant groups compared to native Norwegian women that needs further attention.

### Ethical approval

We have used data from the Medical Birth Registry of Norway, and this registry is approved by the Norwegian Data Inspectorate. The Medical Birth Registry of Norway has approved this study for publication.

## Abbreviations

CI: Confidence Interval;OR: Odds Ratio;PMR: Perinatal Mortality Rate;ENMR: Early Neonatal Mortality Rate;WHO: World Health Organization

## Competing interests

The authors declare that they have no competing interests.

## Authors’ contributions

AE developed the design for the study, with input from the other authors. Statistical computations were carried out by LM with direction and input from JG, who also collected the data. Interpretation of data involved ZN and AE. The manuscript was drafted by ZN and AE. All authors read and approved the final manuscript.

## Pre-publication history

The pre-publication history for this paper can be accessed here:

http://www.biomedcentral.com/1471-2458/13/37/prepub
